# Transcriptome Analysis of the *Capra hircus* Ovary

**DOI:** 10.1371/journal.pone.0121586

**Published:** 2015-03-30

**Authors:** Zhong Quan Zhao, Li Juan Wang, Xiao Wei Sun, Jiao Jiao Zhang, Yong Ju Zhao, Ri Su Na, Jia Hua Zhang

**Affiliations:** 1 Chongqing Engineering Research Center for Herbivores Resource Protection and Utilisation, Southwest University, Chongqing, China; 2 Genetic Engineering and Stem Cell Biology Laboratory, Department of Animal Biotechnology, Faculty of Biotechnology, Jeju National University, Jeju, South Korea; University of Hong Kong, CHINA

## Abstract

**Background:**

*Capra hircus* is an important economic livestock animal, and therefore, it is necessary to discover transcriptome information about their reproductive performance. In this study, we performed *de novo* transcriptome sequencing to produce the first transcriptome dataset for the goat ovary using high-throughput sequencing technologies. The result will contribute to research on goat reproductive performance.

**Method and Results:**

RNA-seq analysis generated more than 38.8 million clean paired end (PE) reads, which were assembled into 80,069 unigenes (mean size = 619 bp). Based on sequence similarity searches, 64,824 (60.6%) genes were identified, among which 29,444 and 11,271 unigenes were assigned to Gene Ontology (GO) categories and Clusters of Orthologous Groups (COG), respectively. Searches in the Kyoto Encyclopedia of Genes and Genomes pathway database (KEGG) showed that 27,766 (63.4%) unigenes were mapped to 258 KEGG pathways. Furthermore, we investigated the transcriptome differences of goat ovaries at two different ages using a tag-based digital gene expression system. We obtained a sequencing depth of over 5.6 million and 5.8 million tags for the two ages and identified a large number of genes associated with reproductive hormones, ovulatory cycle and follicle. Moreover, many antisense transcripts and novel transcripts were found; clusters with similar differential expression patterns, enriched GO terms and metabolic pathways were revealed for the first time with regard to the differentially expressed genes.

**Conclusions:**

The transcriptome provides invaluable new data for a functional genomic resource and future biological research in *Capra hircus*, and it is essential for the in-depth study of candidate genes in breeding programs.

## Introduction

*Capra hircus* is one of the most important livestock animals and the oldest economic domesticated species. Goats have long been used for their milk, meat, hair and skins throughout the world [1–6]. The Dazu black goat, a protected national goat strain in China, has pure black hair and high reproductive performance. The average litter size of multiparous ewes is 2.72. The ovary is a dynamic organ that undergoes structural changes during the mammal reproductive cycle [7–9], which is tightly regulated by a multitude of genes and various endocrine hormones [10–13]. The ovaries play an important role during reproductive processes. There exist many significant differences in the endocrine characteristics and activities of the ovary between mature and immature ewes. In the immature ewe, there is no ovulation, whereas the endocrine pattern varies and ovulation is normal in mature ewe.

Hormone secretion and ovarian follicular development are complex [13,14] and will often change during different developmental stages and in different breeds. Ovarian function is tightly regulated by a large number of genes [13,15,16]. The identification and validation of mRNAs in the ovary at different developmental stages and different breeds [17], however, has been limited. It is needed to identify differentially expressed mRNAs in the ovaries during different developmental stages and different breeds.

In the present study, we investigated the differential expression of mRNAs in different development periods in the ovaries of immature and mature ewe using RNA-seq technology. We used transcriptome sequencing technology to analyse and identify the full repertoire of mRNAs expressed in the ovary during different developmental stages [18,19]. The data provide a large amount of useful information about mRNAs that are related in mammal reproductive biological processes [20–22]. The result will help researchers to further understand the importance of mRNAs in reproductive processes, including hormone secretion and follicular development, and may help to further studies of breeding practices and reproductive regulation in the future.

## Methods and Materials

This study was carried out in strict accordance with the recommendations in the Guide for the International Cooperation Committee of Animal Welfare (ICCAW), which is responsible for animal care and use in China. The experimental conditions were approved by the Committee on the Ethics of Animal Experiments of Southwest University (No. [2007] 3) and the Animal Protection Law in China, and all efforts were made to minimise suffering.

In this study, we performed *de novo* transcriptome sequencing to produce the first transcriptome dataset for the goat ovary using high-throughput sequencing technologies. Our experimental animals were Dazu Black goats which were selected from the Dazu Black Goat Farm at Southwest University, Chongqing, China. We divided our experimental goats into two groups: group A contained sexually mature, barren ewes, and group B contained immature female lambs. Each group contained three goats. When these goats were killed, we collected the ovary immediately.

### Ovary collection and RNA extraction

After pentobarbital sodium 100mg/kg was injected into jugular vein, the muscles were relaxed, the heart and respiratory activities were arrested, we dissect the goats and collected the ovaries immediately and the ovaries were frozen in liquid nitrogen. Total RNA was isolated by a TRIzol Plus RNA Purification Kit (Invitrogen). The concentration and quality of total RNA were determined by an Agilent Technologies 2100 Bioanalyzer.

### cDNA library preparation and Illumina sequencing for transcriptome analysis

To obtain complete gene expression information, a pooled RNA sample that included different developmental stages was used for transcriptome analysis. According to the Illumina manufacturer’s instructions, poly (A)^+^ RNA was purified form 20 μg of pooled total RNA using oligo (dT) magnetic beads and fragmented into short sequences in the presence of divalent cations at 94°C for 5 min. The cleaved poly (A)^+^ RNA was transcribed, and second-strand cDNA synthesis was then performed. After the end repair and ligation of adaptors, the products were amplified by PCR and purified using the PureLink PCR Purification Kit (Invitrogen) to create a cDNA library.

### De novo assembly and function annotation and classification

After removing the reads that contained adaptor contamination, low quality bases and undetermined bases from each of the datasets [23,24], the clean high-quality reads were de novo assembled by a Trinity RNA-Seq Assembler [25].

BLASTX was used to search the NR databases and KEGG database (*E*-value 10^-5^). Blast2go (http://www.blast2go.org/) and InterProScan software [26] were used for Gene Orthology (GO) and KEGG Orthology (KO) annotations of unigenes.

### Digital gene expression (DGE) library sequencing and mapping of DGE tags

Total RNA was extracted from mature and immature ewes using a TRIzol Plus RNA Purification Kit (Invitrogen). Poly (A)^+^ RNA was purified by using oligo (dT) magnetic beads, DGE libraries were prepared by using the Illumina gene expression sample prep kit.

To map the DGE tags, the sequenced raw data were filtered to remove low quality tags, empty tags and tags with only one copy number [27–30]. When annotated, the clean tags were designated as unambiguous clean tags that contained “CATG”, and 21 bp tag sequences were mapped to our transcriptome sequences [31,32]. The number of clean tags for each gene were calculated and normalised to TPM (number of transcripts per million clean tags) for gene expression analysis.

### Quantitative real-time PCR (qRT-PCR) validation

Quantitative real-time reverse transcription PCR (qRT-PCR) was performed to validate the mRNA sequencing data. Total RNA of the two samples corresponding to two growth phases of the Dazu black goat was extracted by TRIzol Plus RNA Purification Kit (Invitrogen). The concentration of each RNA sample was adjusted to 1 μg/μL with nuclease-free water, and 2 μg of total RNA was reverse transcribed in a 20 μl reaction system using the iScript cDNA Synthesis Kit (BIO-RAD). qRT-PCR was performed using the SsoAdvanced SYBR Green Supermix (BIO-RAD) on a CFX96 Real-Time System (BIO-RAD) according to the manufacturer’s protocol, and at least three technical replicates were performed for all genes in each pool. β-actin was used as an internal control gene. The sequences of the specific primers are listed in Table 1. Gene expression difference between two samples was calculated by the 2^-ΔΔCt^ method.

**Table 1:** Primers used in validation experiments.

Unigene Number	Similarity	Abbreviation	qRT-PCR Primer Sequences
Unigene13218	cyclin-D1	CCND1	GCCGAGGAGAACAAGCAG
			GTGTCAGGCGGTGATAGGA
Unigene17906	nucleoporin 54kDa	NUP54	TTCAGAGGAAGAGTGGCTAT
			TTGGGACATCAGTTCATTTA
Unigene27274	interferon alpha-inducible protein 27	IFI27	CCCACCTGGTCCTCTTCTC
			AATCACCGCCTCCTCCTTA
Unigene286	cyclin-B1	CCNB1	AATGTACCCTCCAGAAATCG
			AGGGCGACCCAGACTAAA
Unigene30802	nucleoprotein TPR	TPR	CCATTCCTTTACAGGCTTCA
			CGACGGTATCCTTCAACAT
Unigene7179	intraflagellar transport protein 81	IFT81	GAGATGCCAGAGCAAACA
			TTCCAATCACCAGACCCT
Unigene7507	retinol dehydrogenase 10	RDH10	TGGCTTGTTTCCTTTGAG
			ATTTGGAGTCGTGGGTTT
Unigene7616	zona pellucida glycoprotein 4	ZP4	CCAGTTAATGTCCAGGTTC
			GTAGGAGCGATAGCGTTT
Unigene9759	26S proteasome subunit 11-like	PSMD11	GGTTCTCAGTTGCTTCGGGAGT
			ATGGCATTTGCTGTGGTTCG

## Results

### Illumina sequencing and reads assembly

In this study, the Illumina sequencing method was used to analyse the transcriptome of *Capra hircus*. The transcriptome sequencing data from Dazu black goat have been deposited in the NCBI Sequence Read Archive database (accession number: SRR1556738). A total of 38,771,668 clean reads were generated through Illumina paired-end sequencing and assembled into 150,431 contigs and 80,069 unigenes of the Dazu black goat (Table 2). Those contigs comprised 45.8 Mb of transcriptome sequences with an average length of 305 bp and a N50 length of 479 bp. Those unigenes comprised 49,587,490 total length, with an average length of 619 bp and a N50 length of 1067 bp, including 12,589 distinct clusters and 67,480 distinct singletons.

**Table 2:** Summary of the transcriptome.

Total Number	80,069
Total Length (nt)	49,587,490
Mean Length (nt)	619
N50	1067
Total Consensus Sequences	80,069
Distinct Clusters	12,589
Distinct Singletons	67,480

**Fig 1:**
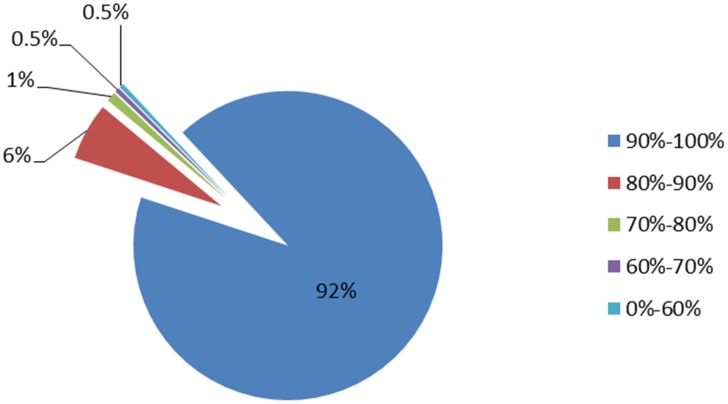
Coverage statistics for genes in *Capra hircus* ovary transciptome.

Gene coverage statistics showed that the number of genes with transcript coverage between 90% and 100% was 73,820 (92%), and genes with coverage between 80% and 90% was 4,652 (6%) (Fig 1). Thus, genes with high coverage accounted for the largest proportion of the mapped genes, indicating that the alignment results were good.

**Fig 2:**
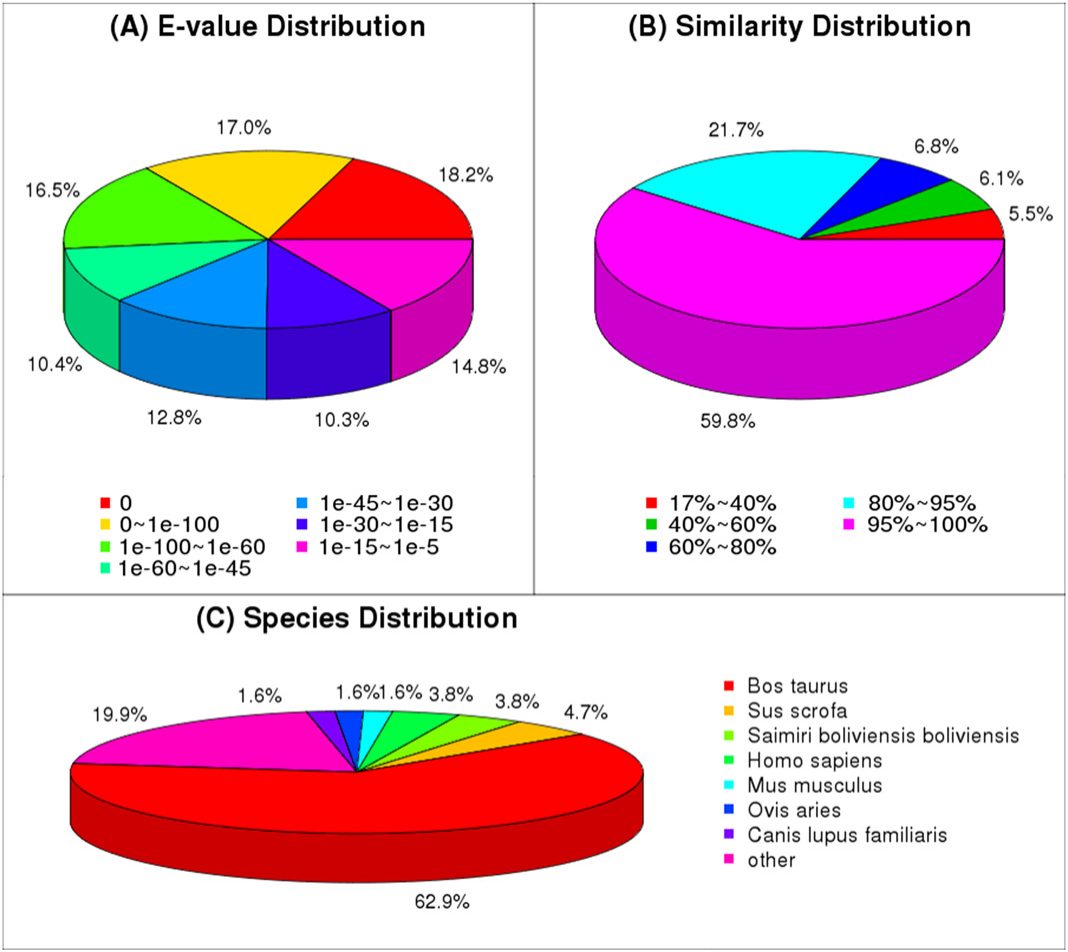
Alignment statistics of the transcriptomes against the nr, nt, and SwissProt databases. A. *E*-value distribution. B. Similarity distribution. C. Species distribution.

The results of a statistical analysis of these new mapped transcripts are shown in Fig 2. The *E*-value distribution showed that matches with an *E*-value of 0 made up the largest portion (18.2%), followed by matches with an *E*-value of (0~1) ×10^-100^(17.0%) and 1×10^-100^~1×10^-60^ (10.4%) (Fig 2A). The similarity distribution showed that transcripts that shared 95%~100% similarity with known sequences accounted for the largest proportion (59.8%), followed by transcripts that shared 80%~95% similarity (21.7%) and transcripts with 60%~80% similarity (6.8%) (Fig 2B). These results indicate that the BLAST results were reliable. The species distribution showed that the majority of matches were with known *Bos Taurus* sequences (62.9%), followed by *Sus scrofa* (4.7%), *Saimiri boliviensis boliviensis* (3.8%), *Homo sapiens* (3.8%), *Ovis aries* (1.6%), *Mus musculus* (1.6%), and other species (19.9%) (Fig 2C). A considerable proportion of the potential unigenes were similar to other species, therefore, further studies are required to improve the goat genome annotations.

### Functional annotation and classification of transcriptome sequences

**Fig 3:**
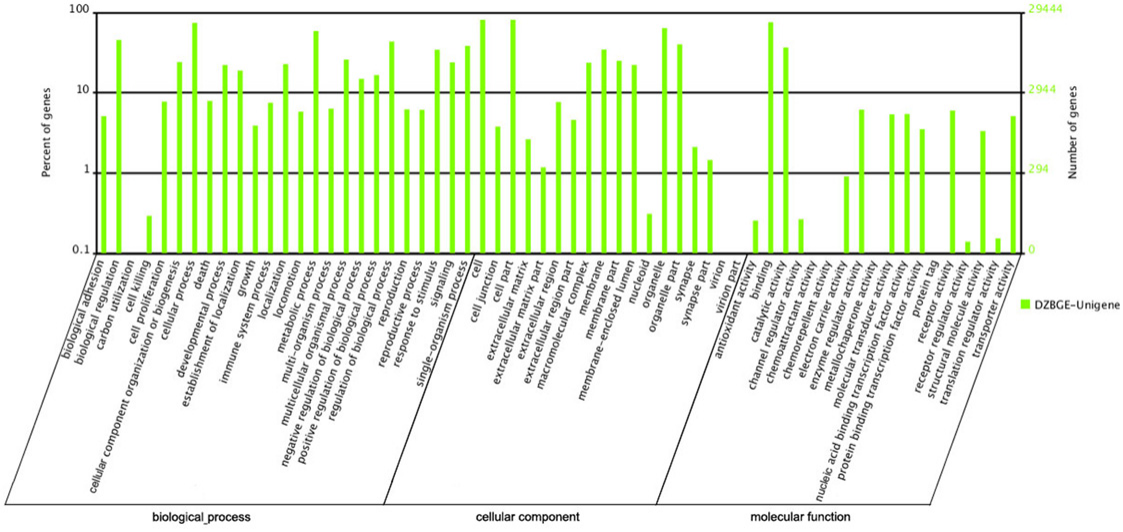
Gene ontology classification of unigenes. Overall, 27,766 unigenes were aligned to at least one GO term. This program categorised 153,403 unigenes in biological processes, 116,403 unigenes in cellular components and 43,931 unigenes in molecular functions.

**Fig 4:**
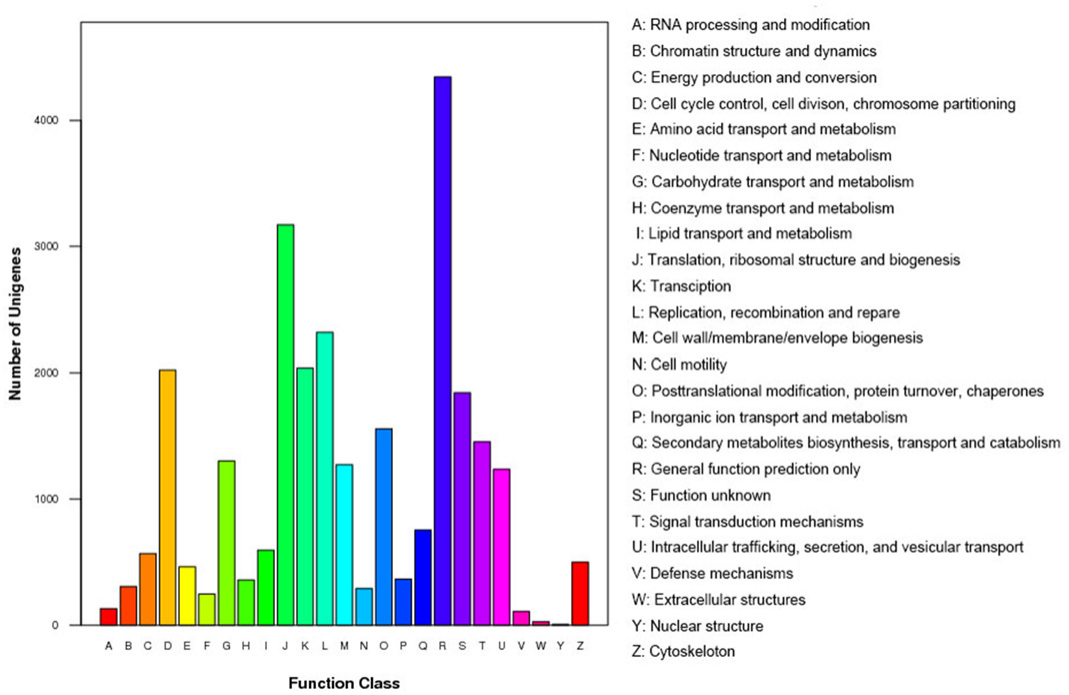
COG annotations of putative proteins. All putative proteins were aligned to the COG database and can be classified functionally into at least 25 molecular families.

**Fig 5:**
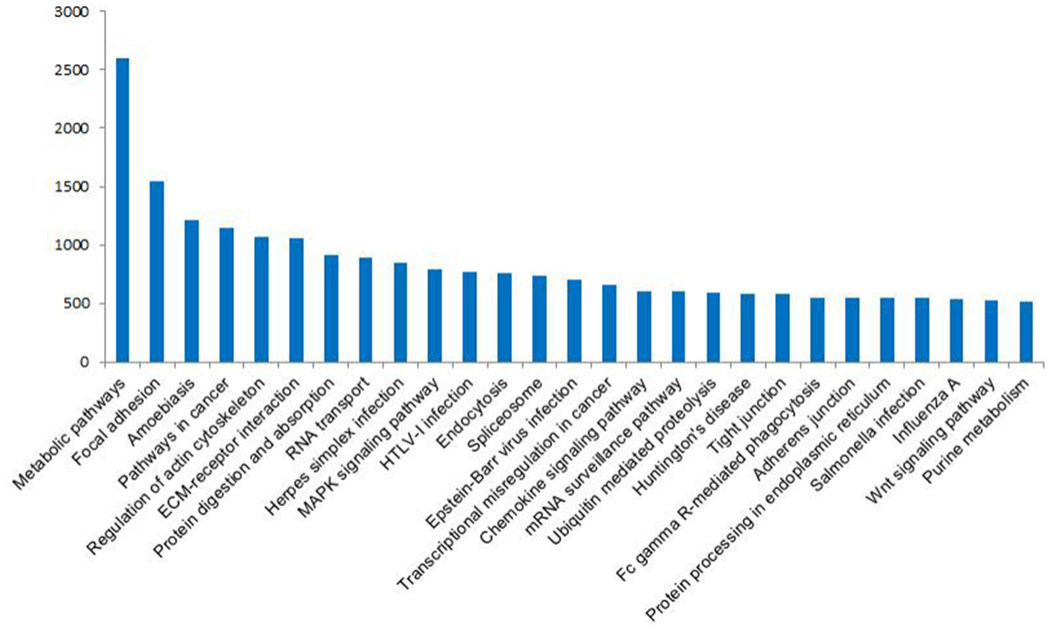
KEGG classifications of unigenes. Overall, 27,766 unigenes were assigned to 258 pathways. The pathways that were mapped by more than 500 unigenes are shown.

We annotated the total 80,069 unigenes and then obtained 64,824 (80.96%) transcript-derived unigenes (Table 3). The sequence similarity search against the non-redundant (NR) protein database revealed that 39,266 out of 64,824 transcript-derived unigenes (60.57%) could be assigned a known function. Gene ontology (GO) annotation (Fig 3) was performed to functionally classify the unigenes that had hits in the NR database, and 29,444 (45.42%) unigenes were annotated with 2,000,299 GO IDs. In the biological process ontology, we found that the most abundant terms annotated to the unigenes were cellular process, metabolism process, and biological regulation, whereas in the cellular component ontology, the most abundant terms were cell, cell part, organelle, and organelle part, and in the molecular function ontology, the most abundant terms were binding and catalytic activity. By mapping the 64,824 unigenes to the Cluster of Orthologous Groups for eukaryotic complete genomes (COG) database [33–35], we found that general function prediction, translation, ribosomal structure and biogenesis were the most frequently represented functional clusters in our transcriptome (Fig 4). We also noted that the replication, recombination and repair cluster and cell cycle control, cell division, chromosome partitioning cluster was frequently represented, indicating the complicated reproduction and reproductive process and its regulation. We also mapped the unigenes to the Kyoto Encyclopedia of Gene and Genomes (KEGG) pathway database [36] and found that 27,766 transcripts mapped to 258 pathways (Fig 5).

**Table 3:** Annotation of unigenes.

Database	Number of Annotated Unigenes	Percentage of Annotated Unigenes
NR	39,266	60.57%
NT	64,571	99.61%
Swiss-Prot	36,910	56.94%
KEGG	27,766	42.83%
COG	11,271	38.28%
GO	29,444	45.42%

### Digital gene expression profiling (DGE) and qRT-PCR validation

Two DGE libraries of goat ovaries, including mature ewes and immature ewes, were sequenced, and 6,191,236 and 5,998,876 raw tags were then generated. After filtered the low quality tags, the total number of clean tags in mature library and immature library were 5,870,617 and 5,658,243, and the percentage of clean tags among the raw tags in mature library and immature library were 94.82% and 94.32%. The sequencing data from two libraries of Dazu black goat have been deposited in the NCBI Sequence Read Archive database (accession number: SRR1561954, SRR1561955). Among the clean tags, the number of sequences that could be mapped to gene tags were 4,818,689 (82.08%) and 4,622,465 (81.69%), and the unique map to gene tags were 3,576,456 (60.72%) and 3,430,932 (60.64%).

To identify the Differentially Expressed Genes (DEGs) associated with the transition from two phases in our DGE, we realigned the short reads that were generated from the two independent libraries related to the two different growth phases back to all transcripts. This process enabled us to evaluate the gene expression abundance by calculating the ∣log_2_Ratio∣ and to estimate the significance of the DEGs between two samples. We observed that no less than 80% of the total clean reads in each library could be mapped uniquely to the all transcripts. In this study, when the False Discovery Rate (FDR) was less than 10^-3^ and the ∣log_2_Ratio∣≥1 between two samples, then the transcripts were considered to be statistically significant DEG.

**Fig 6:**
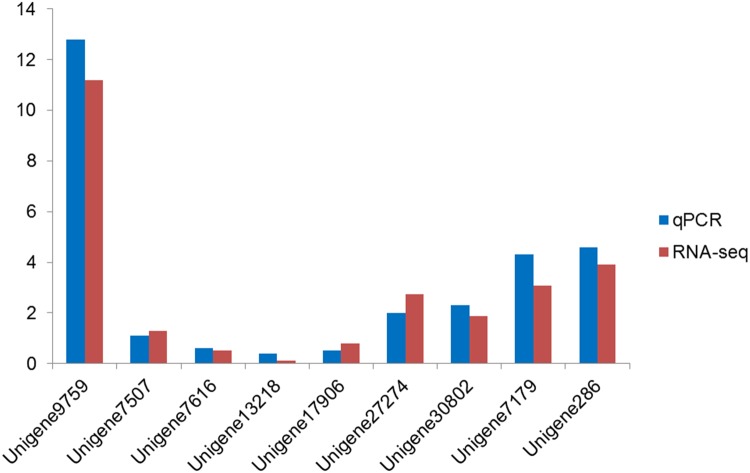
Transcriptome validation. This figure shows that gene expression profiling of the DEGs using qPCR exhibited similar trends to the RNA-Seq samples.

To validate the DEGs with a biological replicate between mature ewes and immature ewes, we selected 9 significant DEGs to perform real time quantitative PCR (qPCR) on the other goat ovary tissues that were previously sampled during the same phase. We found that gene expression profiling of these DEGs using qPCR revealed similar variation trends with RNA-Seq samples (Fig 6), indicating the high credibility of these genes in transcript abundance between mature ewes and immature ewes from different individuals.

### Differentially expressed genes (DEGs) between mature and immature ewe

GO analysis of the transcripts showed that the category associated with reproduction and reproductive process accounted for a large proportion, with the majority of them being involved in the “reproduction” category and there were 1,759 unigenes related to reproduction. Analysing the mature and immature ovary digital gene expression profiling (DGE) data, we found 709 unigenes that were significant different expressed, of which 360 transcripts-derived unigenes from mature ewes were up-regulated compared with immature ewes, and 349 were down-regulated. GO cluster annotation showed that there were 42 transcripts response to reproduction, 36 transcripts response to single organism reproductive process, 23 transcripts response to steroid hormone stimulus, 15 transcripts response to estrogen stimulus, 11 transcripts response to female pregnancy, 9 transcripts response to estradiol stimulus, 6 transcripts response to progesterone stimulus (Table 4). We will continue our research into oocyte maturation. These results provide robust data for further annotation of genes that are related to goat reproduction.

**Table 4:** Reproduction relative DEGs with GO annotation.

Gene Ontology term	DEGs with GO annotation	GO Term ID
response to estrogen stimulus	15	GO:0043627
female pregnancy	11	GO:0007565
single organism reproductive process	36	GO:0044702
embryonic morphogenesis	19	GO:0048598
reproductive process	42	GO:0022414
reproduction	42	GO:0000003
multicellular organism reproduction	21	GO:0032504
multicellular organismal reproductive process	21	GO:0048609
developmental process involved in reproduction	16	GO:0003006
embryo implantation	3	GO:0007566
embryonic organ morphogenesis	8	GO:0048562
regulation of reproductive process	8	GO:2000241
multi-organism reproductive process	19	GO:0044703
development of primary male sexual characteristics	5	GO:0046546
development of primary sexual characteristics	9	GO:0045137
reproductive structure development	10	GO:0048608
reproductive system development	10	GO:0061458
male sex differentiation	5	GO:0046661
embryonic organ development	11	GO:0048568
sex differentiation	9	GO:0007548
negative regulation of reproductive process	3	GO:2000242
cellular process involved in reproduction	12	GO:0048610
positive regulation of reproductive process	4	GO:2000243
ovarian follicle development	3	GO:0001541
embryo development	23	GO:0009790
single-organism reproductive behavior	2	GO:0044704
in utero embryonic development	10	GO:0001701
chordate embryonic development	13	GO:0043009
embryo development ending in birth or egg hatching	13	GO:0009792
reproductive behavior	2	GO:0019098
female gonad development	3	GO:0008585
sexual reproduction	11	GO:0019953
ovulation cycle process	3	GO:0022602
ovulation cycle	3	GO:0042698
positive regulation of viral reproduction	2	GO:0048524
post-embryonic development	2	GO:0009791
development of primary female sexual characteristics	3	GO:0046545
female sex differentiation	3	GO:0046660
regulation of viral reproduction	2	GO:0050792
viral reproductive process	7	GO:0022415
viral reproduction	8	GO:0016032

## Discussion

Traditionally, gene expression analysis of *Capra hircus* has relied mostly on cDNA microarrays, serial analysis of gene expression (SAGE) and expressed sequence tags (EST). However, each of those approaches has some inherent limitations, such as cloning biases, a requirement for prior sequence knowledge, cost, and so on. Thus, the newly developed deep sequencing approaches have significant advantages for investigating the functional complexity of the transcriptome. RNA-Seq is a relatively efficient method for large-scale transcriptomic studies that is constantly being improved. RNA-Seq is used extensively in transcriptomic studies, including gene expression, alternative splicing, determination of non-coding RNA function, and development of SNP or SSR markers. The use of RNA-Seq in studying gene transcriptome information has attracted considerable attention.

In this study, a Dazu black goat ovary pool was constructed by mixing three mature healthy female Dazu black goat ovary samples, to obtain an ovary sequencing library, which after sequencing, yielded 42,377,782 raw reads. After removing low-quality sequences, 38,771,668 clean reads were obtained. The base composition and quality analyses showed that the ratio of Q20 (i.e., the quality of base ≥20) was 98.14% and the GC content was 49.19%, which indicated successful library construction and good sequencing quality. Whole clean reads were assembled into 150,431 contigs and 80,069 unigenes of the Dazu black goat. Those contigs comprised 45.8 Mb of transcriptome sequences with an average length of 305 bp and a N50 length of 479 bp. Those unigenes comprised 49,587,490 total length, with an average length of 619 bp and a N50 length of 1067 bp, including 12,589 distinct clusters and 67,480 distinct singletons.

The function of the expressed genes were annotated by GO and KEGG analyses. A total of 25 GO categories under biological processes were assigned to the transcripts, of which the largest proportion was “cellular process”, followed by “metabolic process” and “biological regulation”. In addition, 1759 unigenes and 1738 unigenes were associated with “reproduction” and “reproductive process”. These results are consistent with the biological characteries and function of the ovary. A total of 18 GO categories under cellular component were assigned to the transcripts of which the largest proportion was “cell” (23873 unigenes). In addition, a significant proportion of the transcripts were associated with membrane components (10185 unigenes), which are important in the physiological activity of the Capra hircus ovary. As an example, endothelial cells of the ovarian capillary and lymphatic system form intracellular channels and a plasma membrane vesicle system, which could play an important role in the tissue fluid and the transport of macromolecular material [6, 9]. A total of 18 GO categories under molecular function were assigned to the transcripts, of which the largest proportion was “binding”. Previous microarray studies showed that the RNA-binding molecular function category accounted for a large proportion in the expressed genes in bovine oocytes [37–39]; thereby confirming the results of the present study that “binding” may plays an important role in the normal physiological activities of goat ovary.

KEGG analysis predicted that the expressed genes were involved in 258 pathways, of which “metabolic pathways” was the most enriched (Fig 5). The metabolism of steroid hormones was significantly important to reproduction and reproductive process. Animal endocrine pathways are maintained in a dynamic balance through the hypothalamus-pituitary-gonadal axis adjustment. Sex steroid hormones include estradiol, testosterone, progesterone and their derivatives [40]. Gonadotropin-releasing hormone (GnRH) secreted in the hypothalamus, can stimulate the complex of gonadal cells in the adenohypophysis and the release of follicle-stimulating hormone (FSH) and luteinizing hormone (LH),which can promote the growth and development of ovarian follicles [6]. “Focal adhesion” was the next most highly enriched. Focal adhesion, a connection function mediated by cells and extracellular matrix (ECM), results in a dynamic cell anchor type of connection, in which the integrins anchor to the ECM [41]. The ECM has comprehensive influence on morphogenesis of embryonic development, including organ formation, or in the adult in maintaining the structure and functional (including immune response and wound repair, etc.), and generally in all life phenomenon. In addition to the “focal adhesion” pathway, the “ECM-receptor interaction” pathway was also enriched. The ECM is a complex matrix of biological macromolecules, such as collagen, fibronectin, laminin, glycosaminoglycans, proteoglycan, adhesive and elastin. The ECM has an important function in various aspects of cell physiological activities, including cell cell shape, structure, function, survival, proliferation, differentiation, migration and so on, by interactions with its surface receptor (ECM receptor). For example, the ECM can send signals to the cells via the surface receptors, and the ECM can send signals to the cytoplasm and nucleus to influence cellular activities or gene expression [42]. In the present study, transcripts associated with the “ECM-receptor interaction” pathway were highly expressed, which suggested that they may be complementary to “focal adhesion”, and both pathways have an important functions in promoting cell adhesion and connection. The enrichment of these two pathways in the transcriptome indicated that cell connections occur extensively in goat ovary. Besides, adherens junction and tight junction were both enriched in the transcriptome (Fig 5). An oocyte mainly communicates with its surrounding cells, such as granulosa and theca cells, through cell adhesion and connection. Numerous small molecular substances that contain metabolites, information, and nutrients that regulate oocyte growth and development are transported through this connection [43, 44]. The “regulation of actin cytoskeleton” pathway was also enriched in the goat ovary transcriptome. Recent studies have shown that actin cytoskeleton also has an important function in the early development of oocyte maturation [45], by assisting in the cytokinesis or the formation of cytoplasmic channels, as well as in the transport of oocyte-specific RNA and proteins. The high expression of transcripts involved in the “regulation of actin cytoskeleton” pathway suggested the existence of dynamic changes in the cytoskeleton structure of goat ovary [46].

Three signaling pathways, mitogen-activated protein kinase (MAPK), chemokine, and Wnt, were also enriched (Fig 5). MAPK is a serine/threonine protein kinase that is available in various signaling pathways, which acts as a common component of signaling transduction in the regulation of cell growth, cell differentiation and cell cycle. The MAPK signaling pathway is also important in eukaryotic signaling networks where it plays a role in passing the upstream signal to the downstream elements [47]. The MAPK pathway is activated when phosphorylation occurs during oocyte mature and metaphaseIIarrest [48]. Wnt is a secreted glycoprotein, which participates in autocrine or paracrine activity. The Wnt protein is important in the regulation of cell proliferation, differentiation, and migration during an organism’s growth and development, and it can determine cell polarity, fate, and proliferation of progenitor cells [49]. Recent studies have shown that the Wnt signaling pathway is necessary to regulate the normal development of the mammalian reproductive system. This pathway is involved mainly in the formation of Mueller’s pipe, control of follicular development, ovulation, and luteinization, and as well as in the establishment of normal pregnancy [50–52]. The enrichment of the MAPK and Wnt pathways in the yak ovary transcriptome is similar to previous studies on oocyte of different species [45, 48, 50, 52], thereby indicating that these two signaling pathways are important in maintaining physiological activity of the ovary. Chemokines play a role in the endometrium of the uterus prior to the implantation of the embryo. The chemokine–receptor signaling at the maternal–fetal interface enables human trophoblast (fetal) to migrate and move into the epithelial region of the endometrium. The bidirectional chemokine mediated signaling between the trophoblast and the maternal endometrium may enable the successful implantation of the embryo. Chemokines also play a functional role in embryogenesis and development of the central nervous system (CNS). The chemokine receptors CXCR4 and CXCR7 and their ligand stromal cell derived factor-1α (SDF-1α/ CXCL12) influence CNS development through the homing of the neuronal precursor cells to their respective target areas of the developing brain [53].

Some enriched pathways, namely, “pathways in cancer”, “amoebiasis” and “Herpes simplex infection”, have no obvious associations with the reproductive function of the ovary. Although these pathways were derived from their corresponding physiological processes, we speculate that some of the genes involved in these pathways may be related to the functions and activities of the ovary. For example, although “pathway in cancer” is related to cancer, an important feature of cancer is proliferation, which is related to the proliferation of germ cells. Thus, some genes involved in the “pathway in cancer” may also be involved in the proliferation and development of ovary-related cells. However, further studies are needed to determine the exact functions of these pathways in the goat ovary.

## Conclusion

In total, 150,431 contigs and 80,069 unigenes were detected in the ovaries of Dazu black goats using transcriptome sequencing technology. Of these, 29,444 and 11,271 unigenes were assigned to Gene Ontology (GO) categories and Clusters of Orthologous Groups (COG), respectively. Searches in the Kyoto Encyclopedia of Genes and Genomes Pathway database (KEGG) showed that 27,766 unigenes were mapped to 258 KEGG pathways. Furthermore, we obtained a sequencing depth of over 5.6 million and 5.8 million tags in the mature and immature ewe, respectively. The transcriptome provides invaluable new data for a functional genomic resource and future biological research in *Capra hircus*, and it is essential for the in-depth study of candidate genes in breeding programs.
